# Clinical analysis of Krukenberg tumours in patients with colorectal cancer—a review of 57 cases

**DOI:** 10.1186/s12957-016-1087-y

**Published:** 2017-01-14

**Authors:** K. Y. Xu, H. Gao, Z. J. Lian, L. Ding, M. Li, J. Gu

**Affiliations:** 1 Department of Surgical Oncology, Beijing Shijitan Hospital and Cancer Centre of Capital Medical University, Beijing, China; 2 Department of Colorectal Surgery, Beijing Cancer Hospital, No. 52, Road Fu Shi, District Haidian, Beijing, China

**Keywords:** Krukenberg tumour, Cytoreductive surgery, Prognostic factors

## Abstract

**Background:**

A Krukenberg tumour (KT) is defined as an ovarian metastasis from a gastrointestinal adenocarcinoma and suggests a terminal condition. This study aimed to identify the prognostic factors affecting the survival of patients with KTs of colorectal origin who receive cytoreductive surgery.

**Methods:**

Medical records of patients who had received cytoreductive surgery and had been pathologically diagnosed with KT of colorectal origin in two centres were reviewed. Information about the patients’ clinicopathological features and follow-up visit were collected. Factors influencing patient survival were analysed.

**Results:**

Fifty-seven patients were included in this study. The median survival time was 35 months. Five-year overall survival was 25%. Patients who had recurrence 2 years after resection of the primary tumour, achieved complete cytoreduction, had metastases confined to the pelvis, had no lymph node involvement, and received systemic chemotherapy had a significantly longer median survival than those who had recurrence at the same time as resection of the primary tumour (*P* = 0.027), received incomplete cytoreduction (*P* < 0.001), had metastases beyond the pelvis (*P* < 0.001), had lymph node involvement (*P* = 0.011), and did not receive systemic chemotherapy (*P* = 0.006) on log-rank test. Less extensive metastatic disease, achievement of complete cytoreduction, and use of systemic chemotherapy were significantly associated with improved prognosis on multivariate analysis.

**Conclusions:**

Cytoreductive surgery may confer survival benefits in patients with KTs of colorectal origin who attain complete cytoreduction and whose metastases are confined to the pelvis and when combined with active systemic chemotherapy.

## Background

Krukenberg tumours (KTs) are defined by the World Health Organization as ovarian carcinomas characterised by the presence of stromal involvement, mucin-producing neoplastic signet ring cells, and ovarian stromal sarcomatoid proliferation [1]. The term has also been applied to metastatic ovarian tumours originating from gastrointestinal adenocarcinomas. Up to 30% of ovarian malignancies are in fact metastatic tumours [2, 3], with the stomach, colorectum, and breast being amongst the most common sites of origin. KTs were reported in 3–14% of women with colorectal cancer [4, 5].

The presence of KTs appears to indicate extensive malignant spread within the abdominal cavity. Indeed, the prognosis for KTs of colorectal origin is so poor that most patients die within 1 year after diagnosis of ovarian metastasis. Chemotherapeutic drugs offering improved tumour response rates in colorectal malignancies generally have low antineoplastic activity in the ovaries, which act as a sanctuary for cancer cells. Surgical intervention may therefore represent a reasonable alternative for the management of ovarian metastatic disease that is insensitive to these agents.

Nevertheless, the role of surgical resection remains controversial in patients with KTs of colorectal origin in light of poor disease prognosis, poor patient tolerance to surgery, low tumour resectability rates, and a high risk of surgical complications. Some studies have shown that resection of metastatic tumours can prolong survival [6, 7], whilst others have found that aggressive surgical therapy offers no benefit for patients with KTs [8, 9]. In this study, we aimed to identify the prognostic factors affecting the survival of patients with KTs of colorectal origin who receive cytoreductive surgery.

## Methods

Patients with a documented diagnosis of malignant neoplasm of the colon, rectum, or ovary between 1994 and 2013 were identified from the medical records of the Capital Medical University Cancer Centre and the Beijing Cancer Hospital. Inclusion criteria for this study included (a) having a confirmed pathological diagnosis of KT of colorectal origin not caused by peritoneal seeding and (b) receiving surgical resection of metastatic tumours. Exclusion criteria included (a) the absence of surgery or histological proof of KT and (b) the validation of an ovarian non-adenocarcinoma metastasis.

All operative records were reviewed, and data pertaining to the primary tumour and ovarian metastatic tumours were collected. These included the main clinical symptoms; the timing of ovarian metastasis (classified as synchronous [detected within 1 year of the primary colorectal cancer diagnosis] or metachronous [detected after more than 1 year]); the extent of surgery (classified as minimal [including salpingo-oophorectomy or oophorectomy only on the macroscopically abnormal side or bilateral salpingo-oophorectomy or oophorectomy] or extensive [including all types of more extensive resections for metastatic tumours such as total abdominal hysterectomy plus bilateral salpingo-oophorectomy, total abdominal hysterectomy plus bilateral salpingo-oophorectomy plus omentectomy, and/or bilateral pelvic and para-aortic lymphadenectomy and/or resection of involved organs]); the completeness of cytoreduction (CC0, no macroscopic residual tumour; CC1, maximal diameter of residual tumour <2.5 mm; CC2, maximal diameter of residual tumour ≥2.5 mm; CC0 and CC1 are described as complete cytoreduction, CC2 as incomplete cytoreduction); the extent of metastatic disease; pathological parameters; follow-up information; and the systemic chemotherapy received.

All patients had clinically detected masses in their ovaries and had received cytoreductive surgery with curative intent, performed by gastrointestinal surgeons in collaboration with gynaecological oncologists, for presumed primary ovarian cancers due to difficulty in differentiation at the time of laparotomy. Patients with peritoneal seeding were additionally treated with early postoperative intraperitoneal chemotherapy. Pathology reports for all patients were reviewed by a pathologist, and the presence of metastatic ovarian cancer of colorectal origin was confirmed. Follow-up time was calculated from the diagnosis of KT to December 2014. Deaths were categorised as events; patients who were still alive at the last follow-up were excluded.

### Statistical analysis

Survival analysis was performed using Kaplan–Meier plots and the difference in survival rates compared using the log-rank test. Variables between groups were compared using the chi-square test. The SPSS computer package (version 15.0, SPSS Inc., Chicago, IL, USA) was used in all analyses. Results were considered statistically significant at *P* < 0.05, and a multivariate analysis by Cox regression model was performed in which *P* values by log-rank test were <0.1 in the univariate analysis.

## Results

Fifty-seven patients who had undergone surgical resection and had been diagnosed pathologically with KTs of colorectal origin were included in the study. Fifteen patients had KTs detected by gastrointestinal surgeons during the course of the treatment for colorectal cancer. Forty-two patients were initially treated by gynaecologists and received operations for ovarian masses, of whom five had colorectal cancer detected during the operation and 37 had histories of colorectal cancer resection. The median follow-up time was 42 months, and the median survival was 35.0 ± 3.5 months (range 6–64 months). Five-year overall survival was 25%.

### Characteristics of primary colorectal cancers and KTs

The mean age at diagnosis of a primary colorectal cancer was 48.2 ± 13.2 (range 23–73) years. The three most common sites of occurrence of a primary adenocarcinoma were the sigmoid colon (19 [33.3%] patients), rectum (11 [19.3%] patients), and ascending colon (nine [15.8%] patients) (Table 1). Lymph node metastasis was confirmed in 36 (63.2%) patients, and retroperitoneal lymph nodes were involved in 16 (28.1%) patients. T3 invasion was seen in 21 (36.8%) patients and T4 in 36 (63.2%) patients.

**Table 1:** Clinical features of 57 patients with KTs of colorectal origin

	Number (*N* = 57)	Percentage
Primary colorectal carcinoma		
Age at diagnosis of primary colorectal carcinoma		
<60 years	43	75.4
≥60 years	14	24.6
Primary tumour site		
Sigmoid colon	19	33.3
Rectum	11	19.3
Ascending colon	9	15.8
Caecum colon	8	14.0
Transverse colon	5	8.8
Descending colon	3	5.3
Multiple sites	2	3.5
Tumour histology		
Well differentiated	19	33.3
Moderately differentiated	21	36.8
Poorly differentiated	11	19.3
Undifferentiated	6	10.5
Depth of tumour invasion		
T3	21	36.8
T4	36	63.2
Primary lymph node metastasis		
Retroperitoneal lymph node metastasis	16	36.8
Non-retroperitoneal lymph node metastasis	20	35.1
None	21	28.1
Ovarian metastatic tumours		
Age at diagnosis of ovarian metastases		
<60 years	40	70.2
≥60 years	17	29.8
Menses		
Pre-menopausal	32	56.1
Post-menopausal	25	43.9
Primary site		
Ovarian mass	18	31.6
Abdominal pain	14	24.6
Abdominal distension	11	19.3
Incidentally discovered during operation	8	14.0
Irregular vaginal bleeding	6	10.5
Timing of ovarian metastasis		
Synchronous	21	36.8
13–24 months	23	40.4
>24 months	13	22.8
Tumour size		
≤10 cm	33	57.9
>10 cm	24	42.1
Ovarian involvement		
Unilateral	21	36.8
Bilateral	36	63.2
Extent of metastatic disease		
*M*_ovary_	28	49.1
M1	14	24.6
M2	15	26.3
Extent of surgery		
Minimal	17	29.8
Extensive	40	70.2
Completeness of cytoreduction		
CC0	26	45.6
CC1	16	28.1
CC2	15	26.3
Systemic chemotherapy		
Yes	34	59.6
No	23	40.4

Note: CC0 and CC1 are described as complete cytoreduction, CC2 as incomplete cytoreduction

*CC0* no macroscopic residual tumour, *CC1* maximal diameter of residual tumour <2.5 mm, *CC2* maximal diameter of residual tumour ≥2.5 mm, *M*_*ovary*_ ovary-only metastasis, *M1* metastasis confined to the pelvis, *M2* metastasis beyond the pelvis

The mean age at diagnosis of KT was 49.3 ± 13.3 (range 24–74) years; 32 (56.1%) patients were pre-menopausal (Table 1). The median interval between the diagnosis of KT and that of a primary colorectal cancer was 17 months. The mean size of KT was 9.7 cm (range 2.2–22.0 cm). Thirty-six (63.2%) patients had bilateral ovarian involvement and 45 (78.9%) patients received bilateral oophorectomy or salpingo-oophorectomy. Contralateral ovaries with a normal appearance were not resected in 12 patients due to patient preference. Minimal surgery was performed on 17 patients (29.8%) whereas extensive surgery, including total abdominal hysterectomy plus bilateral salpingo-oophorectomy, total abdominal hysterectomy plus bilateral salpingo-oophorectomy plus omentectomy, and/or bilateral pelvic and para-aortic lymphadenectomy and/or resection of other involved organs, was performed on 40 patients (70.2%).

Complete cytoreduction including CC0 and CC1 was achieved in 42 (73.7%) patients. Ovary-only metastases (*M*_ovary_) were seen in 28 (49.1%) patients, and metastases beyond the ovaries were seen in 29 (50.9%) patients. The latter were confined to the pelvis (M1) in 14 (24.6%) patients and extended beyond the pelvis (M2) in 15 (26.3%) patients (Table 1).

Amongst patients with *M*_ovary_ or M1, 28 (100%) and eight (57.1%) patients, respectively, received complete cytoreduction. For those with M2, complete cytoreduction was achieved in six patients (40%). Nine (15.8%) patients had postoperative complications, six of whom had metastases beyond the pelvis. Severe complications were seen in three (5.3%) patients (two with an intestinal fistula; one with respiratory complications); all of whom had metastases beyond the pelvis and were >60 years of age.

Thirty-four (59.6%) patients received postoperative chemotherapy (Table 1). All adjuvant chemotherapeutic regimens were fluorouracil-based, generally including mitomycin or levamisole before 2002, and oxaliplatin or irinotecan thereafter. Two patients received bevacizumab for 4–6 cycles; however, this strategy was later abandoned because of cost. Early postoperative intraperitoneal chemotherapy with 5-fluorouracil, mitomycin, cisplatin, or irinotecan was administered for 1–5 cycles in all patients with peritoneal seeding.

### Factors affecting patients’ overall survival (univariate analysis)

Five variables were closely associated with overall survival in the univariate analysis: the timing of ovarian metastasis, the presence of lymph node metastasis, the extent of metastatic disease, the completeness of cytoreduction, and the use of systemic chemotherapy (Table 2).

**Table 2:** Univariate analysis of overall survival in patients with KTs of colorectal origin

	Median	95% confidence interval	*P* value (log-rank)
Age at diagnosis of KT			0.979
<60 years	36	27.546, 44.454	
≥60 years	31	26.394, 35.606	
Menses			0.425
Pre-menopausal	35	27.302, 42.698	
Post-menopausal	38	26.016, 49.984	
Timing of ovarian metastases			0.027
Synchronous	31	3.592, 58.408	
13–24 months	35	28.196, 41.804	
>24 months	54	37.622, 70.378	
Tumour size			0.256
≤10 cm	36	30.511, 41.489	
>10 cm	20	2.402, 37.598	
Status of ovarian involvement			0.570
Unilateral	35	25.783, 44.217	
Bilateral	36	25.828, 46.172	
Extent of metastatic disease			<0.001
*M*_ovary_	54	35.028, 72.972	
M1	35	20.868, 49.132	
M2	13	10.608, 15.392	
Extent of surgery			0.224
Minimal	32	12.493,51.507	
Extensive	38	29.071, 46.929	
Completeness of cytoreduction			<0.001
CC0	56	46.082, 58.200	
CC1	28	10.066, 45.934	
CC2	13	7.951, 18.049	
Systemic chemotherapy			0.006
Yes	47	34.992, 59.008	
No	30	8.932, 51.068	
Depth of tumour invasion			0.117
T3	39	33.234, 44.766	
T4	30	15.296, 44.704	
Lymph node metastasis			0.011
Retroperitoneal lymph node metastasis	18	9.363, 26.637	
Non-retroperitoneal lymph node metastasis	36	19.390, 58.610	
None	39	27.484, 50.516	

Note: CC0 and CC1 are described as complete cytoreduction, CC2 as incomplete cytoreduction

*CC0* no macroscopic residual tumour, *CC1* maximal diameter of residual tumour <2.5 mm, *CC2* maximal diameter of residual tumour ≥2.5 mm, *M*_*ovary*_ ovary-only metastasis, *M1* metastasis confined to the pelvis, *M2* metastasis beyond the pelvis

Survival significantly increased with the disease-free interval between the diagnosis of the primary colorectal cancer and that of the KT (*P* = 0.027), mainly due to the survival difference between patients diagnosed with KT more than 2 years after surgery for removal of a primary tumour and patients with synchronous ovarian metastases by pairwise comparison in the log-rank test (54 months vs 31 months, *P* = 0.008).

Patients with retroperitoneal lymph node metastases had a shorter survival time than those with negative lymph node metastases (18 months vs 44 months, *P* = 0.001) but had a comparable survival time than those with non-retroperitoneal lymph node metastases (*P* = 0.373). By pairwise comparison using the log-rank test, patients with *M*_ovary_ survived longer than those with M1 (*P* = 0.001). Subsequently, patients with M1 survived longer than those with M2 (*P* = 0.014).

The degree of cytoreduction was associated with a significant difference in overall survival (CC0:CC1, 56 months vs 28 months, *P* < 0.001; CC0:CC2, 56 months vs 13 months, *P* < 0.001; CC1:CC2, 28 months vs 13 months, *P* = 0.014). Patients attaining CC0 and CC1 after surgery were considered to have complete cytoreduction; these patients had significantly improved survival compared with those with incomplete cytoreduction (39 months vs 13 months, *P* < 0.001), although the difference did not reach significance in the M2 subgroup by stratified analysis (*P* = 0.253).

The median survival in patients who received systemic chemotherapy was significantly longer than that in patients who did not (47 months vs 30 months, *P* = 0.006).

### Factors affecting patients’ overall survival (multivariate analysis)

A multivariate analysis was performed on five variables: the time to ovarian metastasis, the presence of lymph node metastases, the extent of metastatic disease, the completeness of cytoreduction, and the use of systemic chemotherapy.

**Fig. 1:**
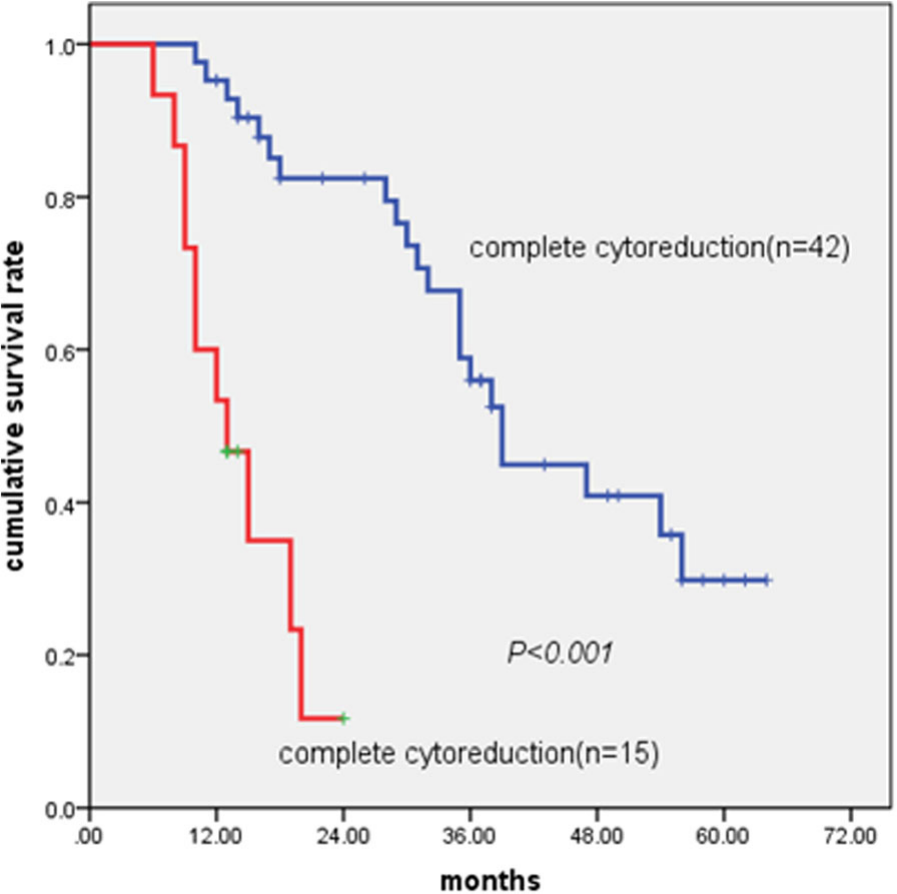
Kaplan–Meier survival curves showing the effect of cytoreduction on patients with Krukenberg tumours of colorectal origin (*P* < 0.001, log-rank test)

**Fig. 2:**
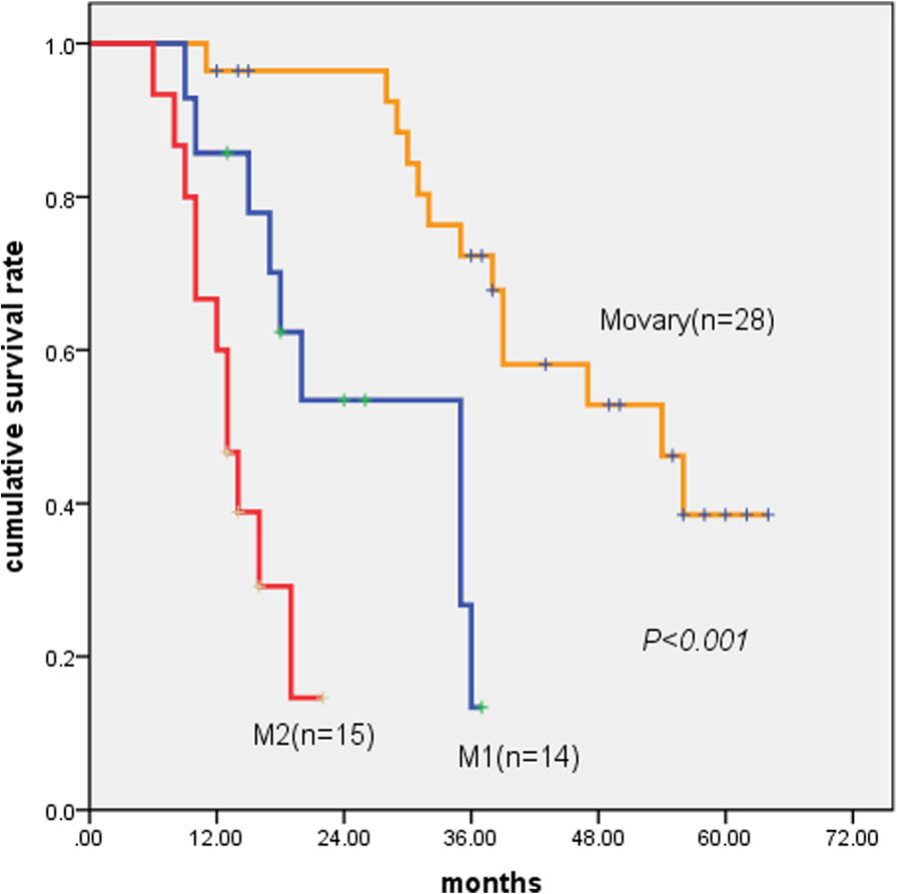
Kaplan–Meier survival curves showing the effect of the extent of metastatic disease on patients with Krukenberg tumours of colorectal origin (*P* < 0.001, log-rank test). *M*_*ovary*_ ovary-only metastasis, *M1* metastasis confined to the pelvis, *M2* metastasis beyond the pelvis

**Fig. 3:**
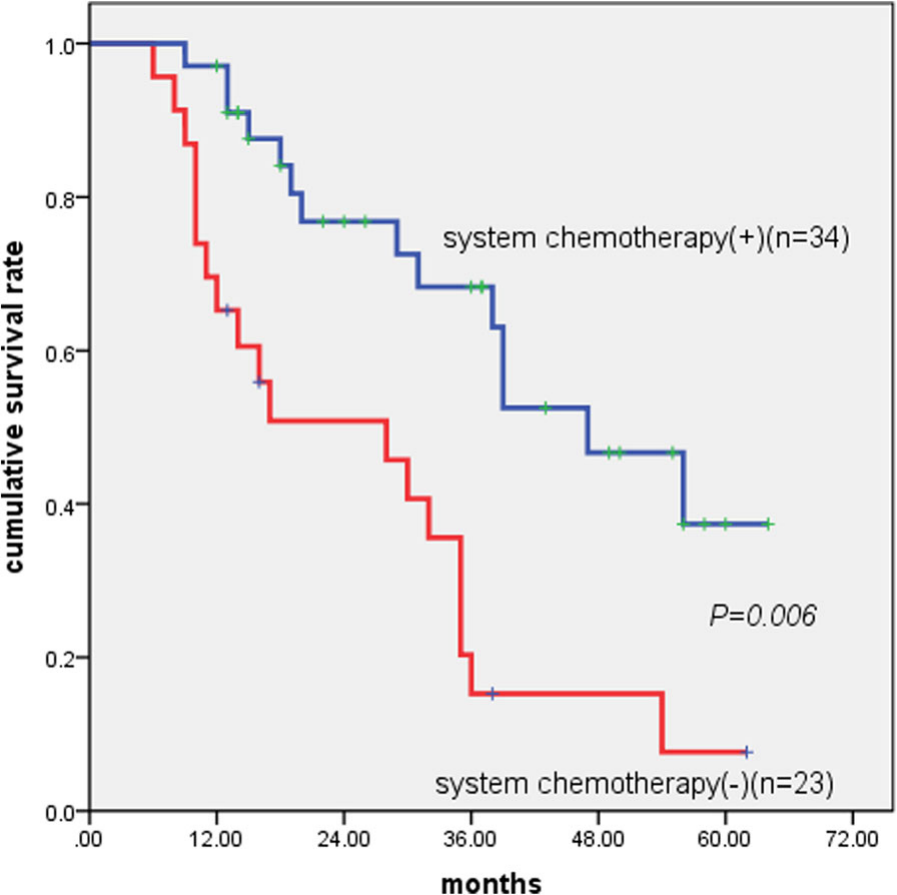
Kaplan–Meier survival curves showing the effect of systemic chemotherapy on patients with Krukenberg tumour of colorectal origin (*P* = 0.006, log-rank test)

Achievement of complete cytoreduction (hazard ratio (HR) 0.135; *P* = 0.001) (Fig. 1), less extensive metastatic disease (HR, 0.287; *P* = 0.029) (Fig. 2), and administration of systemic chemotherapy (HR, 0.345; *P* = 0.012) (Fig. 3) were all independently and strongly associated with improved overall survival. Lymph node metastases had a tendency to be associated with poor prognosis with marginal statistical significance (*P* = 0.061).

## Discussion

Overall, KTs account for 30–40% of metastatic ovarian tumours [10, 11]. The actual incidence of KTs, as seen during autopsy and prophylactic oophorectomy, is much higher than that observed in the clinical setting. The ovary is the second most common intra-abdominal solid organ site of metastasis of colorectal cancer after the liver. At our two centres, KTs occurred in as few as 4.8% of women diagnosed with colorectal cancer during the study period. Women with clinically suspected KTs but who did not undergo surgery were not included in this study.

As shown in previous publications [12, 13], patients in this study were diagnosed with KTs at a median age of 49 years. More than half (32/57, 56.1%) were pre-menopausal, which may be partly because the blood supply to the pre-menopausal ovary increases the risk of metastatic disease [14]. We thus recommend that attention be paid to the ovaries of women with colorectal cancer, particularly those of pre-menopausal women, both at the time of surgery and during follow-up.

Consistent with reports that the incidence of bilateral ovarian metastases ranges from 57 to 70% [13, 15], bilateral ovarian involvement was seen in 63.2% (36/57) of the cohort studied. The American Society of Colon and Rectal Surgeons [16] recommends that oophorectomy be performed in patients suspected or known to have ovarian involvement, either by direct extension or metastasis. If one ovary is found to be positive for metastatic disease, a bilateral oophorectomy should be performed despite limited supporting data [17, 18] because the contralateral ovary has an equal probability of metastatic involvement and may already harbour microscopic metastases.

The prognosis of KTs is generally poor, and in particular, patients with KTs of gastrointestinal origin survive an average of 7–17 months on palliative treatment [19, 20]. Actually considered a potential pattern for peritoneal seeding by Ishii et al. [21], KTs indicate a terminal condition. In the present study, median survival was longer at 35 months (range 6–64 months), possibly as a result of cytoreductive surgery. Death eventually resulted from intra-abdominal tumour progression that was unresponsive to available drugs.

Being a metastatic disease with an inherently poor outcome, surgeons tend to forgo surgical resection for KTs. When surgery is performed, it is frequently intended as palliative care. In contrast, complete surgical resection of metastatic tumours is currently conducted for curative reasons for liver metastasis of colorectal cancer origin [22]. In the current study, cytoreductive surgery was shown to be a significant prognostic factor, and patients with complete cytoreduction achieved a drastic improvement in survival compared with those with incomplete cytoreduction. The greatest benefit of surgery was seen in patients with CC0 (5-year survival, 46.6%; median overall survival, 56 months). Patients with CC1 also survived longer than those with CC2.

Rayson et al. [23] and Morrow and Enker [24] previously drew the same conclusion: complete metastasectomy could result in prolonged survival compared with palliative surgery in patients with KTs of colorectal cancer origin. Additionally, a study in Japan reported that two patients with KTs of colorectal cancer origin who were treated with pelvic exenteration both survived for more than 5 years [25], suggesting that surgery with the intention of removing all gross disease can result in significantly improved survival. Complete cytoreduction plays an important role in patients with KTs of colorectal origin by decreasing the residual tumour burden to an acceptable level in combination with the perioperative use of effective chemotherapeutic agents and new targeted drugs.

Multivariate analysis confirmed the extent of metastatic disease as another indicator of worse prognosis in the present study. Survival was reduced when more sites in the abdominal cavity were invaded by metastatic disease; patients with M2 had the poorest prognosis compared with patients with *M*_ovary_ and M1 and presented with a 5-year overall survival rate of zero. As Miller et al. reported [14], patients with and without peritoneal seeding had a striking difference in overall survival at 5 years (22.6 and 53%, respectively).

Moreover, the extent of metastatic disease was a major determinant of benefit from surgical treatment. In patients with metastases confined to the pelvis, complete cytoreduction can be achieved more easily compared with those with metastases beyond the pelvis (86%:40%, *P* = 0.002, chi-square test). In addition, metastasis beyond the pelvis was associated with a high risk of severe complications, which caused the postoperative death of two patients (not included in this study).

We agree with Elias and colleagues [26] that early surgical intervention for the detection of a small volume of metastasis may optimise survival benefits. Patients with M2, who usually have a poorer general status, are not likely to gain any survival benefit but are prone to severe surgical complications if cytoreductive surgery is undertaken. In our opinion, these factors must be considered before planning surgical intervention for patients with M2.

Systemic chemotherapy was demonstrated as an independent factor of better prognosis in a Cox regression model. Patients who received systemic chemotherapy showed significant improvement in survival compared with those who did not. However, from a stratified Kaplan–Meier analysis, a significant survival benefit was shown only in patients with lymph node metastasis, for which systemic chemotherapy was more intensively recommended.

Limitations of this study included a small sample size, which was influenced by the rarity of the occurrence of KTs and surgeons’ experience, patients’ desire to receive aggressive surgery for its potential benefits, and its retrospective design. However, the study offers important insight into the factors affecting the prognosis of patients with KTs of colorectal origin who receive cytoreductive surgery.

## Conclusions

In conclusion, surgery with curative intent should not easily be abandoned in patients with KTs even if metastases extend beyond the ovaries. Comprehensive preoperative evaluation of the extent of metastatic disease is crucial for treatment planning. Improved survival is possible in patients who attain complete cytoreduction, whose metastases are confined to the pelvis, and who receive active chemotherapy. Future studies should focus on the potentially synergistic effect of surgery and the perioperative administration of cytotoxic and molecular targeted drugs with high response rates.

## Abbreviations

KT: Krukenberg tumour
